# Sexual stigmas among lesbian, gay, and bisexual individuals with problematic internet use and depression

**DOI:** 10.3389/fpsyt.2023.1163032

**Published:** 2023-05-15

**Authors:** Peng-Wei Wang, Yu-Ping Chang, Ching-Shu Tsai, Cheng-Fang Yen

**Affiliations:** ^1^Department of Psychiatry, Kaohsiung Medical University Hospital, Kaohsiung, Taiwan; ^2^Department of Psychiatry, School of Medicine, College of Medicine, Kaohsiung Medical University, Kaohsiung, Taiwan; ^3^School of Nursing, The State University of New York, University at Buffalo, Buffalo, NY, United States; ^4^Department of Child and Adolescent Psychiatry, Chang Gung Memorial Hospital, Kaohsiung Medical Center, Kaohsiung, Taiwan; ^5^School of Medicine, Chang Gung University, Taoyuan, Taiwan; ^6^College of Professional Studies, National Pingtung University of Science and Technology, Pingtung, Taiwan

**Keywords:** problematic internet use, depression, sexual stigma, microaggression, psychological well-being, gay, lesbian, bisexual

## Abstract

**Introduction:**

Sexual stigma causes psychological distress among lesbian, gay, and bisexual (LGB) individuals. LGB individuals are more likely to exhibit both problematic Internet use (PIU) and significant depression than are heterosexual individuals. Whether the severities of sexual stigma varied among LGB individuals with various statuses of comorbid PIU and depression warrants study. The present study aimed to investigate the differences in the experiences of familial sexual stigma (FSS), internalized sexual stigma (ISS), and sexual orientation microaggressions (SOMs) among LGB individuals with various statuses of comorbid PIU and depression.

**Methods:**

In total, 1,000 self-identified LGB young adult individuals participated in the study. The level of PIU was assessed using the Chen Internet Addiction Scale, depression was assessed using the Center for Epidemiological Studies-Depression Scale, the experience of FSS was assessed using the Homosexuality-Related Stigma Scale, the experience of ISS was assessed using the Measure of Internalized Sexual Stigma for Lesbians and Gay Men, and the experience of sexual orientation microaggression was assessed using the Sexual Orientation Microaggression Inventory. The differences in the levels of FSS, ISS, and SOMs among the four groups [i.e., the groups with both PIU and depression (comorbid group), only depression, only PIU, and neither PIU nor depression (neither group)] were investigated using multivariate analysis of covariance.

**Results:**

The results indicated that LGB individuals with comorbid PIU and depression reported higher levels of ISS and SOMs than did those with depression only and PIU only, respectively. Moreover, LGB individuals with PIU or significant depression had higher levels of FSS and SOMs than did those with neither PIU nor depression.

**Discussion:**

The results of this study supported that the experiences of FSS, ISS, and SOMs were significantly associated with various levels of PIU and depression in LGB individuals.

## Introduction

The prevalence of problematic Internet use (PIU) has been increasing worldwide, especially in young people (1). PIU is characterized by an inability to control Internet usage that eventually leads to psychological, social, or work impairment (2); therefore, PIU is closely related to mental health problems (3–5). Online sexual activities are also prevalent in modern times (6) and increase the risk of PIU. Approximately 8–13% of young adults have experienced PIU during their lifetime (7, 8). Given that the Internet has an important role for lesbian, gay and bisexual (LGB) to confirm their sexual identity (9), connect LGB communities (10, 11), find sex partners (12), and engage in cybersex (13), it is hypothesized that LGB individuals are more likely to exhibit PIU than are heterosexual individuals. However, the results of studies investigating whether LGB individuals are overrepresented among individuals with PIU have been conflicting. A study in Sweden indicated that PIU was significantly more prevalent among LGB individuals than among heterosexual individuals (14), whereas a study in seven European countries did not reveal a significant difference in the prevalence of PIU among LGB individuals after psychological distress was controlled for (15). A meta-analysis demonstrated that LGB individuals had a higher risk for common mental disorder such as depressive disorders, alcohol use disorders, anxiety disorders, and suicidality than non-LGB individuals (16). The results of previous studies have supported the necessity of taking mental health problems into consideration when examining the Internet usage and behaviors among LGB individuals.

There has no study examining whether LGB individuals with comorbid PIU and depression experience greater sexual stigma than LGB individuals with PIU only, depression only, and neither PIU nor depression. The experiences of public sexual stigma originating from heteronormality are prevalent among LGB individuals (17). Research found that LGB victims of homophobic bullying exhibited more severe PIU than did non-victims (18). Moreover, according to the minority stress hypothesis, sexual stigma can cause psychological distress among LGB individuals and compromises their mental health (17). Studies have also found that depressive symptoms, hopelessness and loneliness were positively associated with PIU among LGB individuals (19, 20). Therefore, LGB individuals who experience greater sexual stigma are more likely to exhibit both PIU and significant depression is a reasonable hypothesis. Because of the negative impacts of both PIU and depression on the lives of LGB individuals, the association between the types of sexual stigma, comorbid PIU, and significant depression warrants study.

The family is the first social environment in which LGB individuals develop; therefore, familial sexual stigma (FSS) can adversely affect LGB individuals’ identification with their sexual orientation and mental health. When LGB individuals endorse the sexual stigma that they perceived others to have, they may develop internalized sexual stigma (ISS) toward themselves (17). Sexual orientation microaggressions (SOMs) are a more subtle and covert daily demonstration of sexual stigma toward LGB individuals and their sexual orientation than public sexual stigma (21). FSS, ISS, and SOMs are all psychological stressors for LGB individuals (4) and can compromise their mental health (22–24). A study discovered that being victimized by enacted sexual stigma significantly increased the risk of developing PIU (25). Another study reported that problematic social media use was significantly associated with ISS (20). However, the associations of FSS, ISS, and SOMs with PIU warrant further study.

Both individual and environmental factors may influence the relationships among sexual stigma, PIU, and depression among LGB individuals. One of the individual factors is the level of gender non-conformity. Self-identification and self-acceptance of a certain gender role might influence individuals’ behaviors and mental health (26, 27). Gender non-conformity is defined as an individual’s behavioral, cultural, or psychological traits that do not conform to the socially expected traits of the gender associated with the individual’s biological sex (28, 29). Gender non-conformity has been identified as a risk factor for sexuality-related bullying (30, 31) and mental health problems in LGB individuals (32, 33). In addition, outness to family members is significantly associated with LGB individuals’ mental health outcomes (34–36). Therefore, studies should consider the confounding effects of gender non-conformity and outness to family members when examining the differences in sexual stigma among LGB individuals with various levels of PIU and depression.

The present study investigated the differences in the experiences of FSS, ISS, and SOMs among LGB individuals with various levels of Internet use and depression (i.e., with both PIU and depression, only depression, only PIU, and neither PIU nor depression). We hypothesized that LGB individuals with comorbid PIU and depression experience the highest levels of FSS, ISS, and SOMs, followed by those with PIU only or depression only.

## Materials and methods

### Participants

This cross-sectional survey study recruited participants by advertising on popular social media platforms in Taiwan (e.g., Facebook, Twitter, and LINE) and a bulletin board system for LGB individuals, that is, a section of an online message sharing application for LGB individuals in Taiwan, from 1 August 2018, to 31 July 2020.

The inclusion criteria were being (1) a Taiwanese LGB individual and (2) aged between 20 and 30 years. The exclusion criterion was having a major psychiatric disorder (e.g., schizophrenia, cognitive disorders due to traumatic brain injury or other medical conditions) or a substance use disorder, excluding a nicotine use disorder, that might interfere with the participant’s ability to complete the research questionnaire.

Research assistants explained the aims and procedures of the study to eligible participants in the research room. In total, 500 gay and bisexual men and 500 lesbian and bisexual women participated in the study. After providing informed consent, the participants completed paper-based questionnaires. The institutional review board of a hospital affiliated with a medical university approved this study [XXXIRB-F(II)-20180018].

### Measures

#### PIU

This study used the 26-item Chen Internet Addiction Scale (37) to assess the participants’ self-reported PIU severity in the month prior to completing the questionnaire. The participants rated each item on a 4-point scale ranging from 1 (*totally disagree*) to 4 (*totally agree*), with total scores ranging from 26 to 104. A higher total score indicated a higher level of PIU. Studies have verified that the CIAS has acceptable reliability and validity among various populations in Taiwan and have indicated a score of 68 to be the optimal diagnostic cutoff point for PIU (38–40). The Cronbach’s α coefficient of the CIAS in the present study was 0.92.

#### Depression

This study used the 20-item Mandarin Chinese version (41) of the Center for Epidemiological Studies Depression Scale (CES-D (42) to assess the frequency of participants’ depressive symptoms in the month prior to completing the questionnaire. The participants rated each item on a 4-point scale ranging from 0 (*never or very rarely*) to 3 (*always*), with total scores of 0–60. A higher total score indicated greater depressive symptoms. Studies have verified that the Mandarin Chinese version of the CES-D exhibits acceptable reliability and validity among various populations in Taiwan (41, 43). A study in Taiwan determined that a score of 29 or higher could be used to differentiate individuals with and without major depressive disorder (44). In the current study, a score of 29 also indicated the mean CES-D score + 1 standard deviation of all participants. The current study defined participants whose CES-D score was 29 or higher as having significant depression. The Cronbach’s α coefficient of the CES-D in the present study was 0.92.

#### FSS

This study used the 10-item Chinese version of the Homosexuality-Related Stigma Scale (HRSS) (45) to assess the participants’ perceived FSS. Participants rated each item on a 4-point scale ranging from 1 (*strongly disagree*) to 4 (*strongly agree*), with total scores of 10–40. A higher total score indicated a higher level of FSS. Research discovered that the Chinese version of the HRSS has acceptable reliability and validity (45). The Cronbach’s α coefficient of the HRSS in the present study was 0.93.

#### ISS

This study used the 17-item Chinese version (46) of the Measure of Internalized Sexual Stigma for Lesbians and Gay Men (MISS-LG) (47) to assess the participants’ ISS. The participants rated each item on a 5-point scale ranging from 1 (*strongly disagree*) to 5 (*strongly agree*), with total scores of 17–85. A higher total score indicated a higher level of ISS. Research indicated that the Chinese version of the MISS-LG has acceptable reliability and validity (46). The Cronbach’s α coefficient of the MISS-LG in the present study was 0.78.

#### SOMs

This study used the 19-item Chinese version (48) of the Sexual Orientation Microaggression Inventory (SOMI) (49) to assess participants’ SOMs. Participants rated each item on a 5-point scale ranging from 1 (*not at all*) to 5 (*approximately every day/strongly agree*), with total scores of 19–95. A higher total score indicated experiencing more SOMs. Research demonstrated that the Chinese version of the SOMI has acceptable reliability and validity (48). The Cronbach’s α coefficient of the SOMI in the present study was 0.89.

#### Demographic and sexual orientation factors

This study collected the participants’ sex, age, sexual orientation (gay, lesbian, or bisexual), education level, parental education level and marriage status. We assessed self-reported masculinity/femininity with a single item adopted from prior work (50): “Compared with other people of the same age as you, how masculine/feminine would you rate you are?” Each participant’s level of gender role self-identity was self-rated on a 9-point Likert-type scale, with scores ranging from 1 (*extreme femininity*) to 9 (*extreme masculinity*) (51). The responses of the gay and bisexual men were reversely scored. A higher score indicated a higher level of gender non-conformity. We also assessed the level of outness to family with a single item: “How many family members do you let know your sexual orientation?” Participants were grouped into those who reported no or very few family members have known and those who reported some or many family members have known.

### Data analysis

#### Statistical analysis

The sex, age, sexual orientation, FSS, ISS, and SOMs among the four groups (i.e., the groups with both PIU and depression, only depression, only PIU, and neither PIU nor depression) were compared using a chi-square test and analysis of variance. Because the analysis involved multiple comparisons, a *p*-value of <0.004 (0.05/12) was considered to indicate significance. The differences in the levels of FSS, ISS, and SOMs among the four groups were further investigated using multivariate analysis of covariance (MANCOVA). Sociodemographics, sexual orientation, gender non-conformity, outness to family members, and family conditions that differed across various groups were controlled for.

## Results

**FIGURE 1:**
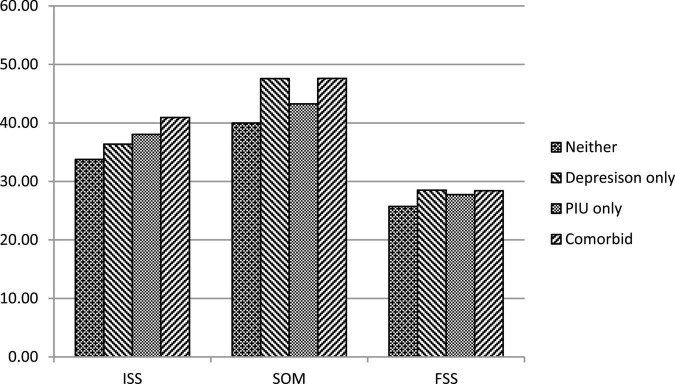
Sexual stigma across the groups with various PIU and depression. PIU, problematic Internet use; Comorbid, the group with both PIU and depression; Depression only, the group with depression only; PIU group, the group with PIU only; Neither: the group with neither PIU nor depression.

In total, 85 (8.5%) participants had comorbid PIU and depression (comorbid group), 157 (15.7%) had PIU only (PIU only group), 113 (11.3%) had depression only (depression only group), and 645 (64.5%) had neither PIU nor depression (neither group). The sex, age, sexual orientation, education level, family conditions, FSS, ISS, and SOMs among the four groups are presented in Table 1. The neither group had a higher proportion of female participants than did the comorbid group. The depression only group had a higher proportion of female participants than did the PIU only group and the comorbid group. No significant differences in age, education level, sexual orientation, gender non-conformity, outness to family members, and parental education level and marriage status were identified among the groups. Significant differences in FSS, ISS, and SOMs were identified among the groups (Figure 1). The results of *post hoc* comparisons revealed that the comorbid group, the PIU only group, and the depression only group experienced greater FSS than did the neither group. The comorbid group experienced greater ISS than did the depression only group and the neither group. The PIU only group experienced greater ISS than did the neither group. The comorbid group and the depression only group had experienced more SOMs than had the PIU only and neither groups.

**TABLE 1:** Sex, age, sexual orientation, education level, family conditions, familial and internalized sexual stigmas, and sexual orientation microaggressions among groups.

	Neither (*n* = 645)	Depression only (*n* = 113)	PIU only (*n* = 157)	Comorbid (*n* = 85)	χ^2^ or *F*	*p*	*Post-hoc* comparison
**Sex, *n* (%)**							
Male (*n* = 500)	314 (62.8)	44 (8.8)	90 (18)	52 (10.4)	13.60	0.004	
Female (*n* = 500)	331 (66.2)	69 (13.8)	67 (13.4)	33 (6.6)			
Age, mean (SD)	24.62 (2.96)	24.04 (3.17)	24.69 (2.93)	25.33 (2.96)	3.04	0.028	
**Sexual orientation, *n* (%)**							
Lesbian or gay (*n* = 570)	367 (64.39)	60 (10.53)	96 (16.84)	47 (8.24)	1.91	0.592	
Bisexual (*n* = 430)	278 (64.65)	53 (12.33)	61 (14.19)	38 (8.83)			
Gender non-conformity	4.43 (1.54)	4.76 (1.57)	4.43 (1.51)	4.68 (1.37)	2.082	0.101	
**Education level**							
High school or below (*n* = 109)	57 (52.29)	16 (14.68)	23 (21.10)	13 (11.93)	8.025	0.046	
College or above (*n* = 891)	588 (65.99)	97 (10.89)	134 (15.04)	72 (8.08)			
**Paternal education level**							
High school or below (*n* = 591)	384 (64.97)	64 (10.83)	93 (15.74)	50 (8.46)	0.338	0.953	
College or above (*n* = 409)	261 (63.81)	49 (11.98)	64 (15.64)	35 (8.55)			
**Maternal education level**							
High school or below (*n* = 660)	418 (63.33)	76 (11.52)	112 (16.97)	54 (8.18)	2.714	0.438	
College or above (*n* = 340)	227 (66.76)	37 (10.88)	45 (13.24)	31 (9.12)			
**Parental marriage status**							
Married and living together (*n* = 709)	465 (65.59)	81 (11.42)	107 (15.09)	56 (7.90)	2.090	0.554	
Separated or divorced (*n* = 291)	180 (61.86)	32 (11.00)	50 (17.18)	29 (9.97)			
**Outness to family**							
Low (*n* = 781)	501 (64.15)	84 (10.76)	125 (16.01)	71 (9.09)	2.681	0.444	
High (*n* = 219)	144 (65.75)	29 (13.24)	32 (14.61)	14 (6.39)			
FSS, mean (SD)^a^	25.74 (6.35)	28.51 (5.85)	27.73 (7.06)	28.40 (5.60)	11.29	<0.001	Comorbid, PIU only, Depression only > Neither
ISS, mean (SD)^a^	33.76 (10.71)	36.35 (12.05)	38.04 (12.63)	40.93 (11.61)	14.53	<0.001	Comorbid > Depression only, Neither PIU only > Neither
SOM, mean (SD)^a^	39.99 (10.56)	47.56 (14.05)	43.25 (10.95)	47.62 (11.91)	24.08	<0.001	Comorbid, Depression only > PIU only > Neither

^a^Data analyzed using analysis of variance.

FSS, familial sexual stigma; ISS, internalized sexual stigma; SD, standard deviation; SOM, sexual orientation microaggression.

The results of the MANCOVA comparing the levels of FSS, ISS, and SOMs among the four groups are presented in Table 2. The FSS, ISS, and SOMs differed significantly among the groups after the effects of sex were controlled for. The neither group exhibited the lowest levels of FSS and SOMs. The comorbid group had experienced more SOMs than had the PIU only group. The comorbid group experienced greater ISS than did the depression only and neither groups. The PIU only group experienced greater ISS than did the neither group.

**TABLE 2:** Comparisons of familial and internalized sexual stigma and sexual orientation microaggressions among groups: multivariate analysis of covariance^a^.

	*F*	*p*	*Post-hoc* comparison
Familial sexual stigma	10.12	<0.001	Comorbid, PIU only, Depression only > Neither
Internalized sexual stigma			Comorbid > Depression only, Neither PIU only > Neither
Sexual orientation microaggression			Comorbid, PIU only, Depression only > Neither Comorbid > PIU only

^a^Controlling for the effects of sex.

## Discussion

The present study discovered that LGB individuals with PIU or significant depression had higher levels of FSS, ISS, and SOMs than did those with neither PIU nor depression; the only exception for this was for ISS, which did not differ significantly between LGB individuals with depression only and those with neither PIU nor depression. Moreover, LGB individuals with comorbid PIU and depression reported higher levels of ISS and SOMs than did those with depression only and PIU only, respectively.

Minority stress theory identifies FSS as distal and SOMs as proximal stressors for LGB individuals (17). LGB individuals who experience these distal and proximal stressors can become hypervigilant because of the expectation of prejudicial events related to sexual orientation and can progressively develop rumination and psychopathology, such as depression (52). Consistently with the results of previous studies (22–24), the present study revealed significant associations of FSS and SOMs with depression in LGB individuals. These results support that intervention programs should be developed to reduce sexual stigma in the public, families, and LGB individuals and to prevent the development of depression among LGB individuals.

The present study also revealed the significant associations of FSS, ISS, and SOMs with PIU. FSS, ISS, and SOMs can cause LGB individuals to be hypervigilant of stigma events; such individuals may reduce their face-to-face contact with others to avoid encountering sexual stigma in the real world. They may, instead, spend considerable time participating in online activities, such as online gaming and chatting, to relieve stress. Although the Internet may provide LGB individuals who experience sexual stigma with a temporary space for relief, PIU may adversely affect their problem-solving abilities and further damage their mental health (4–6). Therefore, PIU and related sexual stigmas among LGB individuals must be evaluated and addressed.

The present study demonstrated that LGB individuals with comorbid PIU and depression reported greater ISS than did those with depression only. The LGB individuals with comorbid PIU and depression had also experienced more SOMs than had those with PIU only. These results indicate that comorbid PIU and depression may present difficulties in the daily lives and mood regulation of LGB individuals who experience severe ISS and SOMs. PIU and depression have a reciprocal relationship; depression predicts the incidence of PIU (53, 54) and PIU predicts the incidence of depression (34). Studies have discovered that common vulnerability and bidirectional cross-causal effects may both contribute to the association between PIU and depression, with common vulnerability likely being more significant than cross-causal effects (54). The results of the current study support that sexual stigma can contribute to the concurrent development of PIU and depression. Both PIU and depression can compromise individuals’ health and their ability to cope with sexual stigma. Therefore, early detection of PIU and depression among LGB individuals and intervention are critical.

The present study found that neither group had a higher proportion of female participants than did the comorbid group, as well as that the depression only group had a higher proportion of female participants than did the PIU only group and the comorbid group. Research has found sex differences in the contents of Internet use and depression. Females are more likely to make intense use of social networks than males, whereas males are more likely to make intense use of massively multiplayer online role-playing games (55). Females also have a higher risk of depressive disorders than males (56). The results of this and previous studies supported that intervention programs for PIU and depression in LGB individuals need to take the role of sex into consideration. This study did not found differences in age, education level, sexual orientation, gender non-conformity, outness to family members, and family conditions across LGB individuals with various PIU and depression. However, further study is needed to replicate the results.

The results of the present study support that sexual stigma should be considered a key health matter for LGB individuals. To reduce FSS, intervention programs should be developed to enhance families’ knowledge regarding sexual orientation, communication between families and LGB individuals, and family support for LGB individuals. To reduce ISS, intervention programs should enhance LGB individuals’ awareness of public sexual stigma, ability to cope with sexual minority stress, and self-affirmation. Regarding SOMs, intervention programs should help LGB individuals develop alternative cognitive and emotional strategies for coping with SOMs and reduce public sexual stigma.

The findings of this study highlight the necessity of developing intervention strategies for preventing and mitigating FSS, ISS and SOM in LGB individuals. In this context, antidiscrimination policies that promise protection from sexual discrimination are instrumental (57). Broadening the understanding of LGB culture and raising awareness of prejudice toward GBM in family and the public constitute crucial steps to reducing stigma surrounding non-heterosexuality (57). The results of the present study also supported that LGB individuals experiencing sexual stigma should be the target of intervention programs for preventing and early detecting PIU and depression to avoid their negative impacts.

### Limitations

This study has some limitations. First, the cross-sectional nature of this study limited the scope of inferences concerning the temporal associations among sexual stigma, PIU, and depression. Second, considering the homogeneity of the sample in terms of age and education level, whether the findings can be generalized to LGB individuals in Taiwan remains unclear. Third, we considered the participants’ biological sex but not their gender identity. Therefore, the effects of the variables on individuals with intersectional identities of sex and gender could not be determined.

## Conclusion

The experiences of FSS, ISS, and SOMs were significantly associated with various levels of PIU and depression in LGB individuals. LGB individuals with comorbid PIU and depression experienced higher levels of ISS and SOMs than did those with depression only and PIU only. LGB individuals experiencing sexual stigma should be the target of intervention programs for preventing and early detecting PIU and depression to avoid their negative impacts.

## Data availability statement

The raw data supporting the conclusions of this article will be made available by the authors, without undue reservation.

## Ethics statement

The studies involving human participants were reviewed and approved by the Kaohsiung Medical University Hospital (KMUHIRB-F(II)-20180018). The patients/participants provided their written informed consent to participate in this study.

## Author contributions

P-WW: analyzing data and writing the original draft. Y-PC and C-ST: review and editing the draft. C-FY: conceptualization, funding acquisition, investigation, and writing the original draft. All authors contributed to the article and approved the submitted version.
